# Genetic variants within microRNA‐binding site of *RAD51B* are associated with risk of cervical cancer in Chinese women

**DOI:** 10.1002/cam4.797

**Published:** 2016-06-23

**Authors:** Dong Hang, Wen Zhou, Meiqun Jia, Lihua Wang, Jing Zhou, Yin Yin, Hongxia Ma, Zhibin Hu, Ni Li, Hongbin Shen

**Affiliations:** ^1^ Department of Epidemiology and BiostatisticsSchool of Public HealthNanjing Medical UniversityNanjingChina; ^2^ State Key Laboratory of Reproductive MedicineNanjing Medical UniversityNanjingChina; ^3^ National Office for Cancer Prevention and ControlCancer Institute and HospitalChinese Academy of Medical SciencesBeijingChina; ^4^ Jiangsu Key Lab of Cancer Biomarkers, Prevention and TreatmentCollaborative Innovation Center for Cancer Personalized MedicineNanjing Medical UniversityNanjingChina

**Keywords:** Cervical cancer, genetic variant, homologous recombinational repair, miRNA‐binding site, *RAD51B*

## Abstract

*RAD51B* plays a central role in homologous recombinational repair (HRR) of DNA double‐strand breaks (DSBs), which is important to prevent genomic instability, a hallmark of cancer. Recent studies suggested that common genetic variants of *RAD51B* may contribute to cancer susceptibility. In this study, we aimed to investigate whether potentially functional variants within miRNA‐binding sites of *RAD51B* are associated with risk of cervical cancer. A total of 1486 cervical cancer patients and 1536 cancer‐free controls were enrolled, and two genetic variants, rs963917 (A > G) and rs963918 (T > C), were genotyped in all participants. Using multivariate logistic regression analyses, we found that G allele of rs963917 conferred lower risk of cervical cancer compared to A allele (adjusted OR = 0.89, 95% CI = 0.80–0.99, *P *=* *0.039). Similarly, rs963918 allele C was associated with a decreased risk for cervical cancer compared with allele T (adjusted OR = 0.84, 95% CI = 0.74–0.94, *P *=* *0.004). Haplotype analyses showed that haplotype GC was also correlated with lower risk (OR = 0.83, 95% CI = 0.73–0.95, *P *=* *0.005) compared with the most common haplotype AT. In summary, our study suggested that miRNA‐binding site genetic variants of *RAD51B* may modify the susceptibility to cervical cancer, which is important to identify individuals with differential risk for this malignancy and to improve the effectiveness of preventive intervention.

## Introduction

*RAD51B*, a known member of the RAD51 paralogs, exerts a key role in homologous recombinational repair (HRR) of DNA double‐strand breaks (DBSs) by promoting the activity of the central recombinase 1. The absence of RAD51B was considered to interrupt formation of the RAD51 nucleoprotein filament, the initial stage of HRR 2. Importantly, unrepaired DSBs can lead to mutations, rearrangements and/or loss of chromosomes, causing genome instability and cancer development 3. Genomic copy number variation on chromosome 14q24.1 that includes *RAD51B* has been frequently detected in pedigrees with Li‐Fraumeni syndrome, and is associated with highly increased risk for squamous cell carcinomas 4. Moreover, the genetic variants of *RAD51B* may contribute to the susceptibility of cancer, as shown in the cases of breast cancer 5, 6, 7, 8, nasopharyngeal carcinoma 9, glioma 10, and cutaneous melanoma 11.

Cervical cancer ranks the third most common malignancy in women worldwide, and human papillomavirus (HPV) is the primary etiologic agent 12. However, although ~80% of women will acquire HPV infection during their lifetime, only a small proportion might progress to invasive cancer 13. Pedigree studies showed that cervical cancer has a significant heritability, supporting a critical role of genetic susceptibility in cervical cancer etiology 14. Even though many studies including two genome‐wide association studies (GWAS) for cervical cancer have identified susceptibility loci in CTLA4, HLA, 4q12, 17q12, etc. 15, 16, 17, these variants explained only a small part of cervical cancer susceptibility. Thus, the missing susceptibility regions are warranted to be further explored.

MicroRNAs (miRNAs) are small single‐stranded noncoding RNA molecules that regulate gene expression by binding to the 3' UTRs of target mRNAs, thus participating in many biological processes, such as cell proliferation, differentiation, apoptosis, and tumorigenesis 18. It has been demonstrated in various studies including ours that genetic variations within miRNA‐binding sites could disrupt miRNA‐mRNA interaction and mRNA expression through changing the binding process 19, 20. Therefore, genetic variants located in the miRNA‐binding sites of *RAD51B* may play critical roles in cervical tumorigenesis.

Using bioinformatics tools (SNPinfo and RNAhybrid) 21, 22, we discovered that two genetic variants (rs963917 and rs963918) in the 3' UTR of *RAD51B* may affect the miRNA‐mRNA interaction. Here, we aimed to evaluate the association between these two potentially functional variants and cervical cancer risk in 1486 patients and 1536 cancer‐free individuals.

## Materials and Methods

### Study participants

This study was approved by the institutional review board of Nanjing Medical University and all participants provided written informed consent before enrollment. The enrollment criteria were described previously 23, 24. In brief, the cases were incident cervical cancer patients and were consecutively recruited from the Nantong Tumor Hospital and the First Affiliated Hospital of Nanjing Medical University from March 2006 to December 2010. All cases were histologically confirmed and those having a history of cancer, metastasized cancer from other organs were excluded. Cancer‐free women controls were randomly selected from a cohort of more than 30,000 individuals who took part in another community‐based screening program for noninfectious diseases conducted in Jiangsu Province. The controls were frequency‐matched to the cases on age (±5 years) and all of them had no self‐reported cancer history. All participants were unrelated ethnic Han Chinese and were interviewed using a standardized questionnaire to collect information on demographic data, menstrual and reproductive history, and environmental exposure history. Individuals who had smoked one or more cigarettes per day for at least 1 year before recruitment or had quit smoking before recruitment were considered as ever smokers; otherwise, subjects were considered as never smokers. After the interview, approximately 5 mL of venous blood was obtained from each participant.

### Variant selection and genotyping

MiRNA‐binding site variants within the 3' UTRs of *RAD51B* were selected using an online bioinformatics tool (http://snpinfo.niehs.nih.gov/snpinfo/snpfunc.htm) 22. A total of 11 candidate variants (rs11622203, rs35948063, rs36103282, rs45538231, rs45568038, rs58791377, rs59490067, rs61658966, rs61985825, rs963917, and rs963918) were predicted to affect miRNA‐mRNA interaction. Then linkage disequilibrium (LD) value (*r* ^2^ < 0.8) and minor allele frequency (MAF ≥ 0.05) in the Chinese Han population (CHB) were applied. As a result, two potentially functional variants (rs963917 and rs963918) were included. RNAhybrid database 21 was also applied to confirm the predicted effect of SNPs on miRNA‐mRNA interaction by calculating minimum‐free energy (MFE).

Genomic DNA was isolated from leukocyte pellets of venous blood using a standard phenol‐chloroform method. Selected genetic variants were genotyped using the Sequenom MassARRAY iPLEX platform (Sequenom Inc. San Diego, CA, USA). Detailed information regarding the primers is presented in Table S1. A series of methods were applied to control the quality of genotyping: (1) genotyping was performed blindly without knowing the status of case or control; (2) two water controls were used as blank controls in each 384‐well plate; and (3) more than 10% samples were randomly selected to be repeated, yielding a concordance rate of >99%.

### Statistical analysis

Distribution differences in demographic characteristics, selected variables, and genotypes between the cases and controls were calculated using the chi‐square test. The associations between genotypes and cervical cancer risk were estimated by computing odds ratios (ORs) and 95% confidence intervals (CIs) in logistic regression models adjusting for age, age at menarche, menopausal status, parity, and smoking status. Age and age at menarche were treated as categorical variables in the models. The chi‐square‐based *Q*‐test was used to assess the heterogeneity between subgroups. All of the statistical analyses were performed with R v.3.2.2 (R Foundation for Statistical Computing, Vienna, Austria) and statistical significance was set at *P *<* *0.05 for two‐sided.

## Results

The characteristics of 1486 cervical cancer cases and 1536 cancer‐free controls were summarized in Table 1. In general, there were no significant differences in the distributions of age and family history of any cancer between the cervical cancer cases and controls (*P *>* *0.05). However, compared with control subjects, the patients with cervical cancer had an earlier menarche (*P *<* *0.001), significantly different menopausal status (*P *=* *0.015), and parity (*P *=* *0.001), and higher proportion of smoking (*P *<* *0.001).

**Table 1:** Demographic and selected variables between cervical cancer cases and controls

Variables	Cases (*N* = 1486)	Controls (*N* = 1536)	*P*
Age, year			
≤50	657 (44.21)	641 (41.73)	0.168
>50	829 (55.79)	895 (58.27)	
Age at menarche, year			
≤16	1069 (71.94)	921 (59.96)	**<0.001**
>16	417 (28.06)	615 (40.04)	
Menopausal status			
Premenopausal	608 (41.70)	598 (38.93)	**0.015**
Natural menopause	769 (52.74)	878 (57.16)	
Unnatural menopause	81 (5.56)	60 (3.91)	
Parity			
0–1	620 (42.29)	731 (48.31)	**0.001**
2	406 (27.69)	405 (26.77)	
>2	440 (30.01)	377 (24.92)	
Smoking status			
Never	1404 (95.77)	1514 (98.57)	**<0.001**
Ever	62 (4.23)	22 (1.43)	
Family history of any cancer			
No	1187 (81.02)	1223 (79.62)	0.335
Yes	278 (18.98)	313 (20.38)	
Histological types			
Squamous cell carcinoma	1380 (92.87)		
Adenocarcinomas	77 (5.18)		
Adenosquamous carcinoma	26 (1.75)		
Others	3 (0.20)		
Stage			
CIN3	10 (0.67)		
I	366 (24.63)		
II	769 (51.75)		
III	187 (12.58)		
IV	45 (3.03)		
Unclassified	109 (7.34)		

The boldface values represent these *P* values were <0.05.

The genotype distributions of genetic variants between cervical cancer cases and cancer‐free controls are shown in Table 2. The genotype frequencies of rs963917 and rs963918 were both in Hardy–Weinberg equilibrium among the controls (*P *=* *0.608 and *P *=* *0.479, respectively). In multivariate regression analyses, G allele of rs963917 showed significantly lower risk of cervical cancer compared to A allele (adjusted OR = 0.89, 95% CI = 0.80–0.99, *P *=* *0.039). Similarly, rs963918 allele C was associated with a decreased risk for cervical cancer compared with allele T (adjusted OR = 0.84, 95% CI = 0.74–0.94, *P *=* *0.004).

**Table 2:** Logistic regression analyses on associations between two SNPs and risk of cervical cancer

SNP	Major/Minor allele	Cases^a^	Controls^a^	OR (95% CI)	*P*	OR (95% CI)^b^	*P* b
rs936917	A/G	446/776/261	432/775/328	0.89 (0.80–0.98)	**0.024**	0.89 (0.80–0.99)	**0.039**
rs936918	T/C	869/530/82	814/596/120	0.82 (0.73–0.92)	**0.001**	0.84 (0.74–0.94)	**0.004**

CI, confidence interval; OR, odds ratio. The boldface values represent these *P* values were <0.05.

a Major homozygote/heterozygote/minor homozygote.

b Adjusted for age, age at menarche, menopausal status, parity, and smoking status.

Furthermore, the LD information of the two genetic variants was calculated using the genotyping data of 1536 controls (*r* ^2^ = 0.329), and then the haplotype analysis was conducted to evaluate the effect of the two variants on cervical cancer risk. As shown in Table 3, haplotype GC (cases: 22.47%, controls: 25.87%) was significantly associated with a decreased risk for cervical cancer (OR = 0.83, 95% CI = 0.73–0.95, *P *=* *0.005) compared with the most common haplotype AT (cases: 55.30%, controls: 51.96%).

**Table 3:** Results of haplotype association analysis

Haplotype^a^	Cases (*N*%)	Controls (*N*%)	OR (95% CI)^b^	*P* b
AT	1637 (55.30)	1589 (51.96)	1.00	–
GC	665 (22.47)	791 (25.87)	**0.83 (0.73–0.95)**	**0.005**
GT	629 (21.25)	633 (20.70)	0.96 (0.84–1.10)	0.592
AC	29 (0.98)	45 (1.47)	0.74 (0.46–1.20)	0.226

The boldface values represent these *P* values were <0.05.

a Haplotypes were composed of the following SNPs in order: rs963917, rs963918.

b Derived from logistic regression with an adjustment for age, age at menarche, menopausal status, parity, and smoking status.

We further conducted stratification analyses on the associations of genetic variants with cervical cancer risk by age, age at menarche, menopausal status, parity, and smoking status. As shown in Table S2, we did not observe significant differences between the subgroups for the associations of rs963917 and rs963918 and cervical cancer risk.

As predicted using SNPinfo, rs963918 T > C may reduce the binding‐free energy of miR18a to *RAD51B* mRNA. This was also confirmed using RNAhybrid database, showing that miR18a has a lower minimum‐free energy (MFE) with C allele (|MFE| = 20.7 kcal/mol) of rs963918 than that with T allele (|MFE| = 22.8 kcal/mol). However, the prediction suggested that miR‐616 might have a slightly higher MFE with G allele (|MFE| = 23.1 kcal/mol) of rs963917 than that with A allele (|MFE| = 22.2 kcal/mol).

## Discussion

HRR pathway is critical for the repair of DSBs, the most harmful type of DNA damage that could lead to genome instability involved in carcinogenesis 25. Many studies have investigated the association of genetic variants in HRR genes (e.g. *BRCA1*/*BRCA2*,* XRCC2*, and *XRCC3*) with various types of cancer, but the results are rather inconsistent 26. The discrepancy may be attributed to differences in sample size, ethnic populations, and study design, as well as the different roles of HRR genes in diverse cancers. However, the risk implication of *RAD51B* variants seems to be an exception, which has been robustly replicated in different studies on breast cancer 5, 6, 7, 8, nasopharyngeal carcinoma 9, glioma 10, and cutaneous melanoma 11, suggesting the significant role of *RAD51B* variants in cancer risk.

Increasing studies including ours have demonstrated that genetic variants located in miRNA‐binding sites of target mRNAs may lead to the deregulation of target gene expression and contribute to the risk of multiple cancers 27, 28, 29, 30, 31, 32. A recent study also found that a functional variant in lnc‐LAMC2‐1:1, a long noncoding RNA (lncRNA), may confer risk of colorectal cancer by affecting miRNA‐binding 33. These studies supported that genetic variants could alter epigenetic regulation (such as miRNA‐mRNA and miRNA‐lncRNA interaction), thereby playing a significant role in carcinogenesis. In this relatively large case–control study, we investigated the association between miRNA‐binding site variants of *RAD51B* and cervical cancer risk among Chinese women. We provided the first evidence that rs963917 and rs963918 were both associated with the risk of cervical cancer. The two genetic variants are located in 14q24.1, and are independent from those published GWAS loci 15, 16, 34. According to bioinformatics analyses, rs963918 C allele may weaken miRNA–mRNA interaction and thus increase the expression of tumor suppressor gene *RAD51B*. The predicted effect is consistent with the protective role of rs963918 allele C in our study. Unfortunately, we did not find such evidence for rs963917.

HPV infection is a necessary but not sufficient cause of cervical cancer. Both host and viral factors can influence clinical outcomes after HPV acquisition 35. As a key event in cervical carcinogenesis, HPV DNA integration into the host genome was suggested to activate oncogenes, inactivate tumor suppressor genes, and increase genome instability 36, 37. Therefore, the efficient repair of DSBs by HRR pathway is especially important for host to prevent HPV integration and disease progression, in which the central role of *RAD51B* can be indirectly reflected by its repeated inactivation by HPV integration 38, 39. Moreover, RAD51B was shown to interact with retinoblastoma (Rb) protein and consequently induced cell apoptosis 40. Given that Rb protein is the target for degradation by HPV E7 oncoprotein to promote cell transformation, a thorough exploration of *RAD51B* function in cervical carcinogenesis will help to understand the complex interaction between the virus and host.

This study has several limitations. First, we conducted only one stage case–control study. Studies of large sample size across different populations are needed to validate the current results. Second, although we found bioinformatics evidence, the biological effects of these two genetic variants on *RAD51B* expression and function were not further investigated in this study. Third, we could not collect cervical specimens to detect HPV infections among the participants. It remains unknown on the interaction between *RAD51B* variants and a particular HPV type.

In conclusion, our study provided two genetic biomarkers (rs963917 and rs963918) associated with cervical cancer, which would help to identify individuals at differential risk for this malignancy. Further studies with different ethnic background and biological function analyses are warranted to uncover the mechanism of *RAD51B* in cervical cancer development.

## Conflict of Interest

The authors declare no conflicts of interest.

## Supporting information


**Table S1.** Information of primers for Sequenom MassARRAY iPLEX.
**Table S2.** Stratified analyses on association between two SNPs and cervical cancer risk.
