# Supplementary material for: Genetic variants within microRNA‐binding site of RAD51B are associated with risk of cervical cancer in Chinese women
Source: Cancer Med. 2016 Jun 23;5(9):2596–601. doi: 10.1002/cam4.797 (PMC5055154; doi:10.1002/cam4.797)
Supplement: Supplementary file 1 — Table S1. Information of primers for Sequenom MassARRAY iPLEX. Table S2. Stratified analyses on association between two SNPs and cervical cancer risk. [file CAM4-5-2596-s001.docx]

Table S1. Information of primers for Sequenom MassARRAY iPLEX.

| Variants | Sequence (5'-3') |
| --- | --- |
| rs963917 | F: ACGTTGGATGTATGACCAGAACAAGGGAGC |
|  | R: ACGTTGGATGCCAATACCATATGGTCGAAC |
|  | E: gtAAGGGAGCAGCCTGA |
| rs963918 | F: ACGTTGGATGTCAAGGGAGCCTGTTTTGTC |
|  | R: ACGTTGGATGAGGAGTGGCCTTGGATGTTC |
|  | E: ggGTTATACTCAATTCATTTGTTCAT |

Table S2. Stratified analyses on association between two SNPs and cervical cancer risk.

| Characteristics | rs963917 | | | | *P*_het_^b^ | rs963918 | | | | *P*_het_^b^ |
| --- | --- | --- | --- | --- | --- | --- | --- | --- | --- | --- |
|  | Case | Control | OR(95% CI)^a^ | *P*^a^ |  | Case | Control | OR(95% CI)^a^ | *P*^a^ |  |
| Age, year |  |  |  |  | 0.255 |  |  |  |  | 0.274 |
| ≤50 | 189/341/126 | 175/333/132 | 0.96(0.81-1.13) | 0.642 |  | 374/246/36 | 333/273/35 | 0.91(0.75-1.10) | 0.329 |  |
| >50 | 257/435/135 | 257/442/196 | 0.85(0.74-0.98) | 0.021 |  | 495/284/46 | 481/323/85 | 0.79(0.68-0.93) | 0.003 |  |
| Age at menarche, year |  |  |  |  | 0.499 |  |  |  |  | 0.992 |
| ≤16 | 321/562/185 | 257/463/201 | 0.87(0.76-0.99) | 0.036 |  | 632/377/56 | 498/349/70 | 0.84(0.72-0.97) | 0.018 |  |
| >16 | 125/214/76 | 175/312/127 | 0.94(0.78-1.13) | 0.525 |  | 237/153/26 | 316/247/50 | 0.84(0.68-1.03) | 0.093 |  |
| Menopausal status |  |  |  |  | 0.793 |  |  |  |  | 0.765 |
| Premenopausal | 182/313/112 | 158/309/130 | 0.87(0.74-1.03) | 0.118 |  | 359/217/31 | 316/244/35 | 0.85(0.7-1.04) | 0.112 |  |
| Natural menopause | 232/405/130 | 257/435/186 | 0.89(0.77-1.03) | 0.125 |  | 453/266/47 | 469/324/82 | 0.82(0.7-0.96) | 0.013 |  |
| Unnatural menopause | 23/42/16 | 17/31/12 | 0.85(0.47-1.52) | 0.575 |  | 38/40/2 | 29/28/3 | 0.82(0.4-1.66) | 0.583 |  |
| Parity |  |  |  |  | 0.267 |  |  |  |  | 0.798 |
| 0~1 | 171/330/117 | 192/384/155 | 0.93(0.79-1.09) | 0.383 |  | 348/238/31 | 375/303/49 | 0.85(0.71-1.02) | 0.078 |  |
| 2 | 130/209/67 | 118/194/93 | 0.81(0.66-1.00) | 0.045 |  | 248/136/22 | 223/147/35 | 0.82(0.65-1.03) | 0.085 |  |
| >2 | 137/228/74 | 113/186/77 | 0.93(0.76-1.14) | 0.489 |  | 261/150/27 | 205/136/34 | 0.84(0.67-1.05) | 0.132 |  |
| Smoking status |  |  |  |  | 0.381 |  |  |  |  | 0.113 |
| No | 418/734/249 | 426/760/327 | 0.89(0.80-0.99) | 0.030 |  | 824/498/77 | 799/590/119 | 0.83(0.73-0.93) | 0.002 |  |
| Yes | 20/33/9 | 6/15/1 | 1.31(0.56-3.23) | 0.539 |  | 33/26/3 | 15/6/1 | 1.78(0.73-4.89) | 0.230 |  |

^a^Adjusted for age, age at menarche, menopausal status, parity and smoking status (excluded the stratified factor in each stratum);

^b^*P*-value for the heterogeneity test.
